# The consequences of COVID-19 lockdown for formal and informal resource utilization among home-dwelling people with dementia: results from the prospective PAN.DEM study

**DOI:** 10.1186/s12913-021-07041-8

**Published:** 2021-09-22

**Authors:** Maarja Vislapuu, Renira C. Angeles, Line I. Berge, Egil Kjerstad, Marie H. Gedde, Bettina S. Husebo

**Affiliations:** 1 Department of Global Public Health and Primary Care, Centre for Elderly and Nursing Home Medicine, University of Bergen, Årstadveien 17, 5009 Bergen, Norway; 2 NORCE Norwegian Research Centre AS, Bergen, Norway; 3 NKS Olaviken Gerontopsychiatric Hospital, Erdal, Norway; 4 Haraldsplass Deaconess Hospital, Bergen, Norway; 5 Department of Nursing Home Medicine, Municipality of Bergen, Bergen, Norway

**Keywords:** COVID-19, Dementia, Caregiver, Resource utilization, Formal care, Informal care

## Abstract

**Background:**

COVID-19 isolated home-dwelling people with dementia (PwD) from home care services, respite care, and daytime activities. We aimed to investigate the consequences of these restrictions on informal (family, friends) and formal (homecare staff) resource utilization among co-residing (e.g., spouses) and visiting caregivers (e.g., children).

**Methods:**

105 PwD (≥65 years old) and their caregivers were included in the prospective PANdemic in DEMentia (PAN.DEM) study, which was initiated when the ongoing stepped-wedge, cluster randomized LIVE@Home.Path trial (*N* = 438) was temporarily halted due to the pandemic. Primary outcome was change in resource utilization assessed by the Resource Utilization in Dementia Care (RUD) instrument in pre- (12 Dec. 2019 to 11 Mar. 2020) and during the lockdown periods (20 April 2020 to 15 May 2020). Degree of cognitive impairment was assessed by Mini-Mental Status Examination (MMSE), and physical functioning and independent living skills by Physical Self-Maintenance Scale and Lawton Instrumental Activities of Daily Living Scale. Associations between informal and formal care utilization, socio-demographics, and clinical variables were assessed by descriptive statistics and Ordinary Least Squares models (OLS).

**Results:**

Mean age for PwD was 81.8 years; 61% were female; 45.6% lived alone, and the mean MMSE score was 20.8 (SD ± 3.7). PwD with co-residents (44%) were younger (78.4 years) than those who were living alone (84.5 years; *P* < 0.001). During the first 2 months of lockdown, PwD missed on average 20.5 h of formal care in a month (P < 0.001) leading to an approximately 100% increase in informal care, which was particularly pronounced in personal hygiene (6.9 vs. 11.4 days in a month, *P* < 0.001) and supervision (9.2 vs. 17.6 days in a month; P < 0.001). Visiting caregivers increased by 1.9 days (SD ± 11.5), but co-residing caregivers increased their number of days providing ADL by approximately 7 days per month (β = 6.9; CI, 0.39–13.1, *P* < 0.05) after adjusting for PwD and caregiver demographics and clinical variables. Decrease in home nursing care was particularly visible for PwD living alone (− 6.1 vs. -1.3 h per month, *P* = 0.005). Higher cognitive function (β = − 0.64, CI, − 1.26 – 0.02, *P* = 0.044) was associated with reduction in home nursing service during the lockdown.

**Conclusion:**

The care situation for PwD changed dramatically in the early phase of the COVID-19 pandemic, especially for those living alone who received less support from homecare services and visiting caregivers. For future crises and the forthcoming post-pandemic period, health authorities must plan better and identify and prioritize those in greatest need.

**Trial registration:**

ClinicalTrials.gov; NCT04043364.

## Background

Health systems must be able to adapt effectively to changing conditions with limited resources. Early on in the COVID-19 pandemic, one of society’s most pressing issues became the provision of adequate care and treatment for home-dwelling people with dementia (PwD). To protect the lives of older adults, a restrictive policy was rigorously implemented by the Norwegian government and all municipal-based services such as daycare, respite care, and even daily homecare support were closed (as of 12 March 2020) [1, 2].

Countries worldwide adopted different lockdown policies. The U. K and Sweden, implemented a “shielding policy” (people in vulnerable groups were asked to stay at home),to protect the elderly over 70 years of age [3–5]. China, Norway and Germany, implemented social distancing measures for the entire population [4–6]. In Germany during the lockdown, incident diagnoses decreased 17–26% among 2.45 million older patients, including those with a dementia diagnosis [6]. This is concerning as dementia diagnosis is a prerequisite for dementia-tailored home-based care services [7]. A UK study (*n* = 14,891) showed individuals with chronic illnesses to be twice as likely to cancel their health care appointments than the general population. They were also twice as likely to need increased care hours. Authors suggest that a significant proportion of people having one or more chronical illnesses were deprived of essential care during the lockdown [8].

In Norway, 101,000 people (1.9% of the population) have dementia [9], of whom 70,000 (69%) are living at home and provided with treatment and care by formal homecare services and informal caregivers; the latter are often co-residing (e.g., spouses) or visiting (e.g., children). To support older adults staying safely, independently, and for a longer time in their own homes, the Norwegian welfare state administers social and care services by the municipalities [10]. This work includes medication aid, wound dressing, help with personal hygiene, and social, physical, and mental activities outside the home. These services are offered to about 50% of home-dwelling PwD [11].

The service disruption during COVID-19 raises concerns about the direct consequences for PwD and their caregivers, because social distancing and isolation may decrease self-care ability and exacerbate informal caregiver burden and stress, as well as worsen the behavioral and psychological symptoms of dementia (BPSD) such as agitation, anxiety and psychosis [12–15]. A UK cross-sectional survey of COVID-19 related social support closures and their effects on 569 participants indicates that the reduction in formal service hours increased levels of anxiety in older people and PwD and in addition, the mental well-being of informal caregivers was reduced [16]. Other authors suggest that the pandemic requires even more informal caregiving compared to the pre-pandemic burden, and previous research has shown that co-residency is a common denominator for the scope of informal care utilization and high caregiver burden [17, 18]. However, none of these studies have investigated the relationship between formal and informal care during the pandemic-induced reductions in formal services. Clearly there is a need for more investigation about the consequences of COVID-19 for different caregiver groups.

To investigate how COVID-19 affected the lives of home-dwelling PwD and their caregivers, our research group initiated the prospective PAN.DEM study nested within the ongoing trial LIVE@Home.Path [19, 20]. This study aimed to investigate the consequences of the initial phase of COVID-19 restrictions on informal and formal resource utilization among co-residing and visiting caregivers. We hypothesize that 1) the ratio of formal vs. informal care changed during the COVID-19 lockdown, with an increase in informal care; 2) the provision of informal care increased more among co-residing caregivers compared to visiting caregivers; 3) the prioritization of formal care services changed in order to support alone living PwD to compensate for decreased family support.

## Methods

### Participants and enrolment

**Fig. 1:**
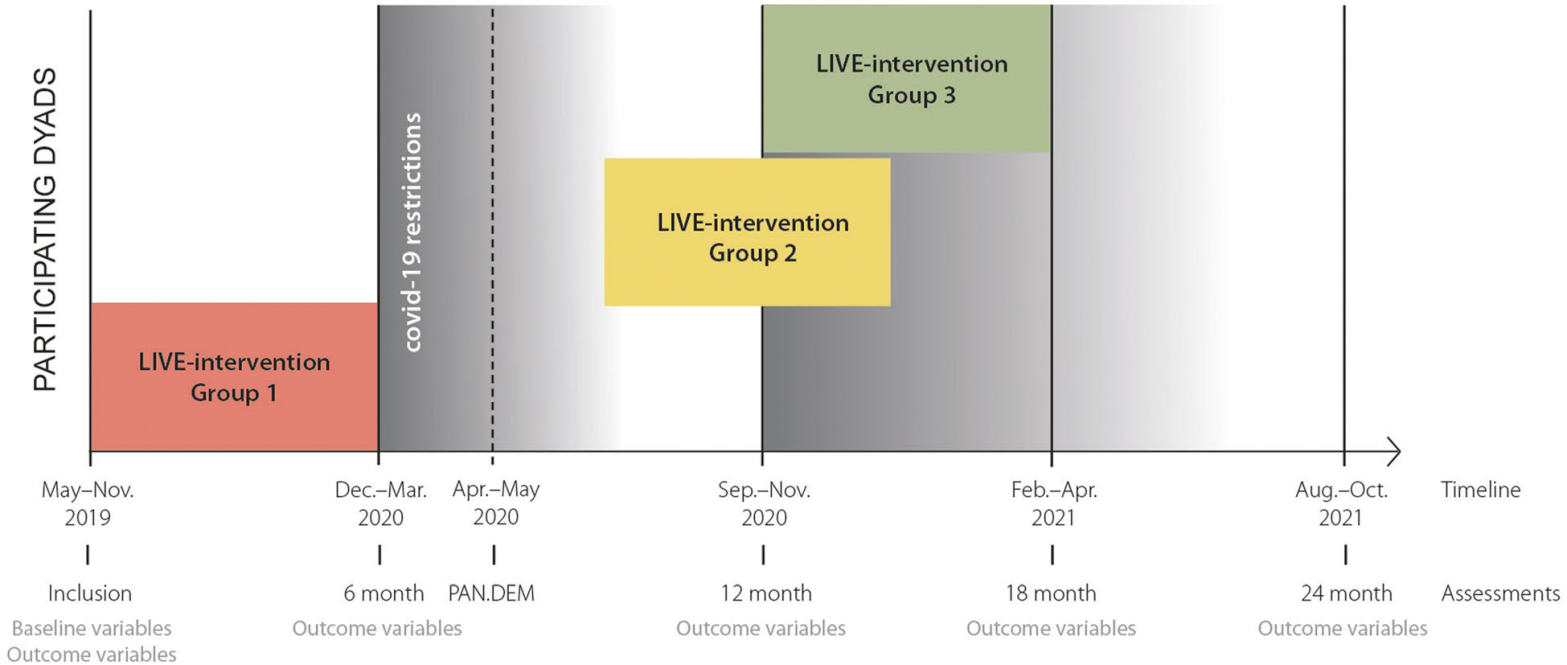
The LIVE@Home.Path trial timeline, including Pandemic in Dementia (PAN.DEM) study

LIVE@Home.Path is a 24-month, stepped wedge randomized controlled trial for home-dwelling PwD and their informal caregivers (dyads) to investigate the effect of a multicomponent LIVE intervention on resource utilization in municipal dementia care (tentative study period May 2019 – April 2021). LIVE is the acronym for the intervention comprising Learning, Innovation, Volunteers, and Empowerment [20]. Home-dwelling PwD were eligible for inclusion if they were ≥ 65 years old, diagnosed with dementia according to the standardized protocol, a Mini-Mental Status Examination (MMSE) score 15–26 [21] or a Functional Assessment Staging scale (FAST) score 3–7 [22], lived together with an informal caregiver (co-residing), or physically meeting family members/friends at least once a week (visiting) [19]. The stepped wedge design implies that all dyads will receive the 6 months intervention, but the timing is determined by randomization (Fig. 1).

The COVID-19 restrictions temporarily halted the implementation of the intervention in the LIVE@Home.Path. To investigate how the pandemic affected the dyads, our research group initiated the prospective PAN.DEM cohort study consecutively including caregivers by semi-structured telephone interviews from 20 April 2020 to 15 May 2020 [19]. Pre- lockdown data was collected in between 12 December 2019 and 11 March 2020. PAN.DEM contained core assessment instruments from LIVE@Home.Path in addition to questions about how the lockdown affected the dyads’ everyday life [19]. Data were collected using tablet computers and stored securely on the internal data server at the University of Bergen, Norway, to ensure data privacy and quality.

### Measurements

The primary outcome of this study was the change in formal and informal caregiving as assessed by The Resource Utilization in Dementia (RUD), which is a measurement developed by Wimo et al. [23] that assesses the frequency and number of hours during the last 30 days spent on informal care in supervision, including assisting basic self-care tasks (ADL) and instrumental tasks (IADL) that require more extensive planning skills. ADL includes assistance with functional mobility, toileting, bathing, hygiene, and eating. IADL includes care tasks such as shopping for groceries, preparing meals, undertaking household chores, and doing laundry [23]. In addition to informal care, RUD also measures the frequency and number of hours of formal care during the last 30 days (home nursing, home help, meals on wheels, transportation, and daycare). The instrument is validated for the assessment of resource utilization in nursing homes and for home-dwelling PwD [23–25]. To measure the change in pre- and post-pandemic regulation outcomes, we divided the caregivers according to their living status (co-residing or visiting).

### Covariates

To investigate the potential changes of informal care before and after lockdown among co-residing and visiting caregivers and informal care use, we included caregiver demographics (age, gender, level of education, and employment status). In addition, we measured cognitive impairment by MMSE (score range 0–30): a score ≤ 23 indicates considerable cognitive impairment [26]. Instrumental Activities of Daily Living Scale (IADL) was used to assess the level of functioning [27] and the Physical Self-Maintenance Scale (PSMS) was used for the level of physical self-care [28]. For both IADL (range 8–31) and PSMS (range 6–30), higher scores indicate reduced ability to self-care in activities of daily living [27, 28]. The clinical global impressions of change (CGIC) was used to assess the perceived change in the total situation by the primary caregiver during the lockdown [29], with the caregivers’ answers trichotomized to improved/worsened/no change.

### Statistical analysis

Descriptive statistics were presented by mean, standard deviation (SD) and frequencies (%). To consider normality in the continuous variables, histograms and QQ plots were used as a first approach in addition to the Shapiro-Wilk test [30]. Differences between caregivers groups were evaluated by the Mann-Whitney U test for non-normally distributed continuous variables, and Pearson χ^2^ tests for categorical variables. Welch’s unequal variance t-test was used to compare the change in formal and informal care utilization between groups of caregivers. Wilcoxon Signed-Rank test was used to compare change between time use in pre- and lockdown period in single samples. Missing data in descriptive statistics was handled by pairwise deletion.

Due to the structure of our data, ordinary least squares (OLS) regression was used to examine the association between the change in informal care frequency and residency of caregiver – before and after the lockdown. We used an interaction term between a time dummy indicating the value 0 and 1 for pre- and post-lockdown outcome, and a dummy for co-residency, thus comparing co-residential caregiver*lockdown to visiting caregiver*pre-lockdown. We used the Akaike information criterion (AIC) to determine the best-fit model within the range of included covariates. As caregivers and PwDs are nested within municipalities (*n* = 3), we included municipalities as dummy variables to control of time-invariant effects. Finally, we checked the main model for robustness to exclude the possibility of autocorrelation. The β-coefficient of statistically significant covariates are presented with 95% confidence intervals (CIs). All tests were two-sided, and a *P*-value < 0.05 was considered statistically significant. The data were analyzed with Stata/SE, 16 (Stata, College Station, TX).

## Results

**Fig. 2:**
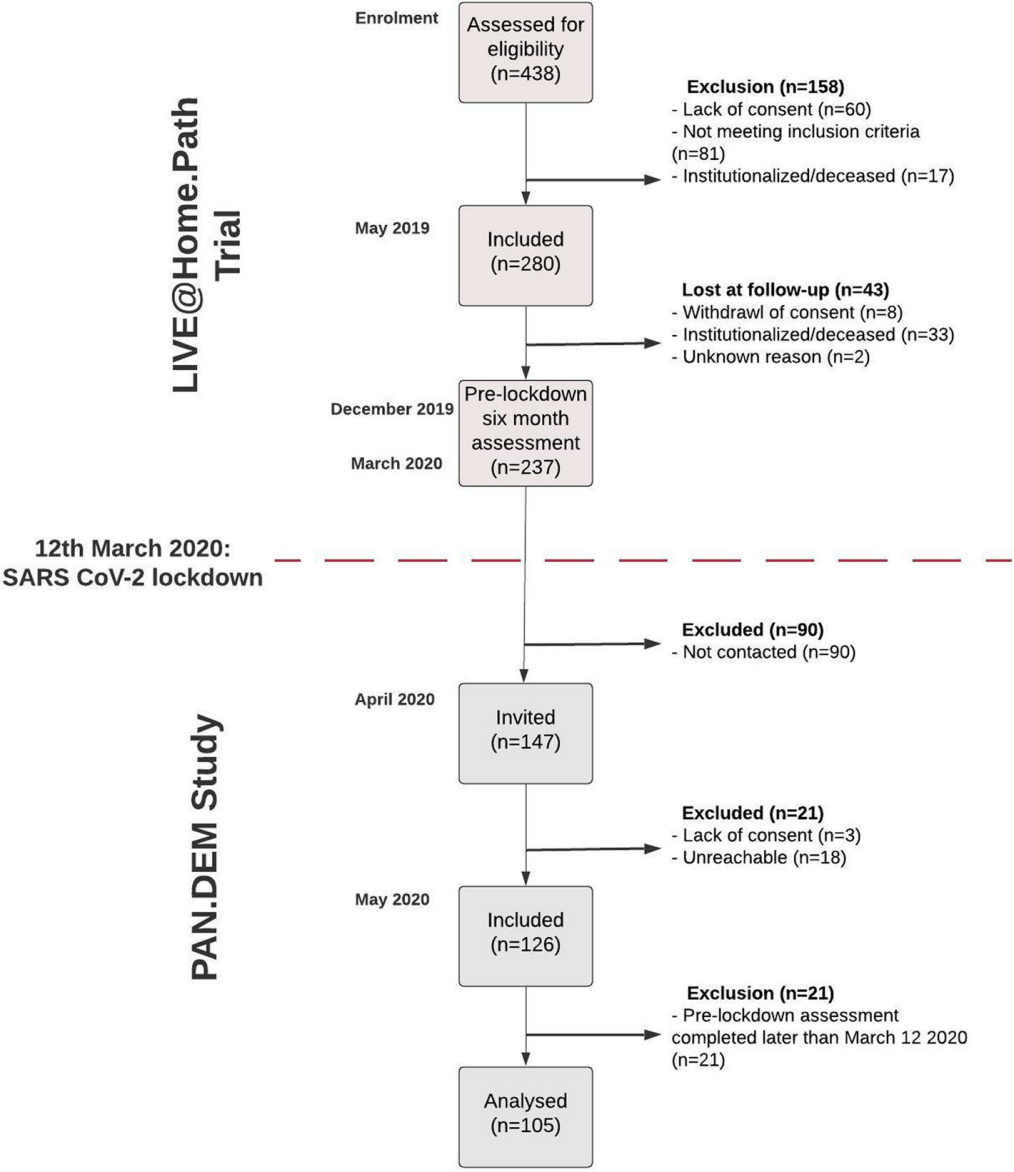
Study design and participant flow

438 dyads were assessed for eligibility to participate in the LIVE@Home.Path trial. Of these, 158 were excluded because of institutionalization (*n* = 17), not meeting the inclusion criteria (*n* = 81), or lack of consent (*n* = 60). In May 2019, 280 dyads were enrolled in the study, and after 6 months, 237 completed the pre-lockdown assessment, of which 126 dyads were included in the subsequent PAN.DEM study. We excluded 21 dyads from our analyses because their pre-lockdown assessment was completed after the COVID-19 restrictions were effectuated. In total, there was a resulting study sample of 105 dyads (Fig. 2).

The mean age of the PwD was 81.8 years (SD ± 6.9); 61% were female; 45.6% lived alone; the majority had mild or moderate degrees of dementia (mean MMSE score 20.8, SD ± 3.7) (Table 1). PwD co-residing with informal caregivers (*N* = 46, 43.8%) were younger (78.4 years, SD ±6.0) than those living alone (84.5, SD ± 6.4, *P* < 0.001).

**Table 1:** Pre-lockdown characteristics for caregivers and people with dementia, by residency; (*N* = 105)

Variables	All (***n*** = 105)	Co-residing caregivers (***n*** = 46)	Visiting caregivers (***n*** = 59)	Difference, p-value^a^	Missing data (n)
**PwD characteristics**					
Age, mean (SD)	81.8 (6.9)	78.4 (6.0)	84.5 (6.4)	**< 0.001**	–
Gender, female, n (%)	64 (61)	17 (37.0)	47 (79.7)	**< 0.001**	–
Living alone, yes, n (%)	47 (45.6)	0 (0)	47 (79.7)	**< 0.001**	2
MMSE, mean (SD)	20.8 (3.7)	20.8 (3.4)	20.7 (3.6)	0.896	4
IADL, mean (SD)	21.8 (5.1)	22.3 (5.2)	21.4 (5.1)	0.411	1
PSMS, mean (SD)	11.7 (3.6)	11.6 (4.0)	11.8 (3.3)	0.499	2
**Caregivers characteristics**					
Age, mean (SD)	65.5 (12.1)	74.6 (9.8)	58.3 (8.4)	**< 0.001**	–
Gender, female, n (%)	69 (65.7)	31 (67.4)	38 (64.4)	0.749	–
Higher education, yes, n (%)	67 (63.8)	28 (62.2)	39 (68.4)	0.513	3
Working, yes, n (%)	48 (45.7)	4 (91.3)	44 (74.6)	**< 0.001**	–
Number of additional care providers, n (%)				0.173	–
- 0 (no additional)	17 (16.2)	10 (21.7)	7 (11.9)		
- 1+ (one or more additional caregivers)	88 (83.8)	36 (78.3)	52 (88.1)		
Relationship, n (%)				**< 0.001**	–
- Spouse	44 (41.9)	42 (91.3)	2 (3.4)		
- Child	59 (56.2)	4 (8.7)	55 (93.2)		
- Other	2 (1.9)	–	2 (3.4)		
Primary caregivers’ contribution, n (%)				**< 0.001**	–
- 1–20%	2 (1.9)	–	2 (3.4)		
- 21–40%	7 (6.7)	–	7 (11.9)		
- 41–60%	16 (15.2)	2 (4.4)	14 (23.7)		
- 61–80%	14 (13.3)	1 (2.2)	13 (22.0)		
- 81–100%	66 (62.9)	43 (93.5)	23 (39.0)		

*N* = total sample, *n* = number of participants

Data are mean (SD) and number (%)

^a^Tested with The Mann-Whitney U test for nonnormally distributed continuous variables, and Pearson χ^2^ tests for categorical variables; *MMSE* Mini Mental Status Examination (range 0–30), higher scores indicate better cognition, *IADL* Instrumental Activities of Daily Living Scale (range 8–31), lower scores indicate better functioning, *PSMS* Physical Self-Maintenance Scale (range 6–30), lower scores indicate better functioning

The mean age of the informal caregivers was 65.5 years (SD ± 12.1); 65.7% were female; 48.3% were co-residing with the PwD. Of caregivers, 56% were children and 41.6% were spouses; 66% considered themselves to be the primary caregiver (Table 1). The mean age for co-residing caregivers was 74.6 years (SD ± 9.8), while the mean age for visiting caregivers was 58.3 years (SD ±8.4, P < 0.001). More caregivers in both groups were female (co-residing 67.4%, visiting caregivers 64.4%).

### The change in formal and informal care from pre-lockdown to lockdown

As assessed by CGIC, the caregiver situation before versus after lockdown worsened for 67.6% of a PwD’s relatives; 27.6% reported no change in their condition; 4.8% reported improvement (Table 2). During lockdown, nine informal caregivers were laid off from their employment. 60% of the caregivers reported that the pandemic had consequences for formal care services and that support was reduced or not delivered at all by municipalities or hospitals (e.g., daycare, tour groups, respite services, and outpatient appointments at hospitals). In addition, 22% of caregivers reported that they had delayed or cancelled services for the PwD due to the pandemic.

**Table 2:** Experience during the lockdown for the 105 dyads, reported by the caregivers

	All (***n*** = 105)	Co-residing caregivers (***n*** = 46)	Visiting caregivers (***n*** = 59)	Difference, ***p***-value^a^	Missing data (n)
Have health care services been influenced by pandemic, yes	63 (60.2)	27 (58.7)	36 (61.0)	0.810	–
Have you or person with dementia self-canceled services^b^, yes	21 (22.1)	9 (21.4)	12 (22.6)	0.887	10
Are you temporarily laid off due to the COVID-19 restrictions, yes	9 (8.6)	2 (4.35)	7 (11.86)	**< 0.001**	–
The overall situation compared to immediately before the pandemic to lockdown:				0.600	–
- Worse	71 (67.6)	15 (32.6)	42 (71.2)		
- Better	5 (4.8)	2 (4.4)	3 (5.1)		
- No change	29 (27.6)	15 (32.6)	14 (23.7)		

Results are presented in n (%)

^a^Tested with The Mann-Whitney U test for nonnormally distributed continuous variables and Pearson χ^2^ tests for categorical variables

^b^Includes primary and secondary health care services

Table 3 shows a significant change in the number of users and average hours of formal caregiving when the pre-lockdown numbers are compared to lockdown numbers, with a reduction from 23.7 h per month (SD ± 29.6) to 3.6 h per month (SD ±10.0). The number of PwD receiving home nursing decreased from 65.7 to 34.3% (*P* < 0.001), corresponding to a reduction of 4.2 h per month (SD ±10.3). Home help (e.g., doing laundry and cleaning) was reduced from 38 to 18% of the PwD representing a loss of 0.5 h per month (SD ± 2.0, *P* = 0.011), and daycare centers were closed for all participants (40.0 to 0%; − 15.5, SD ±25.8, *P* < 0.001). The overall informal care increased in ADL (6.9 vs. 11.4 days/month, P < 0.001) and supervision (9.2 vs. 17.6 days/month, P < 0.001).

**Table 3:** Formal health care service utilization and the provision of informal care for the total sample before and during the lockdown, *N* = 105

Formal care	Number of users pre-lockdown, yes n (%)	Hrs/mth Mean (SD)	Number of users during the lockdown, yes n (%)	Hrs/mth Mean (SD)	Mean change (SD), hrs/mth	Difference, ***p***-value^a^
Home nursing	69 (65.7)	7.4 (10.8)	36 (34.3)	3.3 (9.6)	−4.2 (10.3)	**< 0.001**
Home help	38 (36.2)	0.8 (1.9)	18 (17.1)	0.3 (0.9)	−0.5 (2.0)	**0.011**
Daycare center	42 (40.0)	15.5 (25.8)	0	–	−15.5 (25.8)	**< 0.001**
Food delivery	8 (7.6)	NA	6 (5.7)	NA	NA	0.881
Transportation (care related)	21 (20)	NA	0	NA	NA	**< 0.001**
Total^b^	80 (76.2)	23.7 (29.6)	38 (36.2)	3.6 (10.0)	−20.5 (29.0)	**< 0.001**
**Informal care**		**Days/mth** **Mean (SD)**		**Days/mth** **Mean (SD)**	**Mean change (SD), days/mth**	
IADL^c^	101 (96.2)	18.0 (12.0)	97 (92.4)	17.4 (12.1)	−0.6 (11.5)	0.058
ADL^d^	39 (37.1)	6.9 (11.3)	52 (49.5)	11.4 (13.7)	4.5 (13.7)	**< 0.001**
Supervision^e^	42 (40)	9.2 (13.0)	76 (72.4)	17.6 (13.5)	8.4 (16.1)	**< 0.001**

*N* = total sample, *n* = number of patients

^a^Paired sample t-test

^b^Home nursing, home help and daycare center

^c^includes care tasks like taking medicine, grocery shopping, and doing administrative tasks

^d^includes care tasks like toileting, hygiene, and eating

^e^includes supervision in daily tasks and preventing dangerous situations, also calling to ensure well-being

NA – Not applicable

Table 4 presents formal and informal care change by comparing pre-lockdown and lockdown by residency of caregiver groups. Both groups showed a significant decrease in home nursing (*P* = 0.002 and < 0.001) and daycare (P < 0.001), and an increase in informal supervision time (*P* = 0.004 and 0.001). PwD supported by visiting caregivers lost more home nursing care hours (6.1 h per month) than PwD who were co-residing (1.3 h per month, *P* = 0.005). Informal care increased more in the co-residing group compared to the visiting group (+ 7.8 days/month vs. + 1.9 days/month, *P* = 0.035).

**Table 4:** Formal and informal care use before and during the lockdown by residency of caregivers (*N* = 105)

	Co-residing caregivers (***n*** = 46)			Visiting caregivers (***n*** = 59)			***P***-value^a^
	Pre- lockdown	Lockdown	P-value^b^	Pre- lockdown	Lockdown	*P*-value^b^	
**Formal care hours in a month**							
Home nursing	2.0 (4.3)	0.8 (3.1)	**0.002**	11.7 (12.4)	5.3 (12.1)	**< 0.001**	**0.005**
Home help	0.4 (1.3)	0.01 (0.3)	0.235	1.1 (2.3)	0.5 (1.1)	**< 0.001**	0.287
Daycare	15.1 (25.6)	0	**< 0.001**	15.8 (26.2)	0	**< 0.001**	0.879
Total formal care	17.4 (26.5)	0.8 (3.3)	**< 0.001**	28.6 (31.1)	5.8 (12.7)	**< 0.001**	0.265
**Informal care days in a month**							
IADL	26.6 (8.9)	27.4 (7.5)	0.962	11.3 (9.6)	9.5 (8.7)	0.412	0.280
ADL	10.3 (13.8)	18.1 (14.1)	**0.050**	4.3 (8.1)	6.2 (10.9)	0.322	**0.035**
Supervision	13.0 (14.8)	20.9 (13.4)	**0.004**	6.2 (10.6)	15.0 (13.1)	**0.001**	0.773

*N* = total sample, *n* number of patients, *IADL* Instrumental Activities of Daily Living, *ADL* Basic Activities of Daily Living

^a^Welch’s unequal variance t-test was used to compare the change between caregiver groups by residency

^b^Wilcoxon Signed-Rank test was used to compare change between time use in pre- and lockdown period

### The change in informal care comparing pre-lockdown with lockdown by caregiver characteristics

Table 5 presents the OLS regression. The baseline model [1] shows that the lockdown led to an increase in ADL informal care. Visiting caregivers increased by 1.9 days (SD ± 11.5), but co-residing caregivers increased their number of days providing ADL by approximately 7 days per month (β = 6.9; CI, 0.39–13.1, *P* < 0.05) after adjusting for PwD and caregiver demographics (age, gender) and clinical variables (BPSD, MMSE, and ADL functioning). We extended model 1 by including the variable additional caregivers (model 2). This variable counts additional caregivers to the primary caregiver who is either co-residing or visiting PwD. In model 2 the interaction term still holds, with a small reduction in the beta coefficient (β = − 6.6, CI, − 0.36-12.93, *P* < 0.05). In model 3 we included a robustness test to correct for autocorrelation. The interaction term remains unchanged (β = − 6.8, CI, − 0.16-13.54, P < 0.05).

**Table 5:** OLS regressions of the number of informal days after the lockdown, *N* = 105

Covariates	Model 1				Model 2				Model 3			
	Coef.	95% CI lower	95% CI upper	*P*	Coef.	95% CI lower	95% CI upper	*P*	Coef.	95% CI lower	95% CI upper	*P*
**Time**												
Lockdown^a^	0.86	−3.38	5.10	0.690	1.08	−3.05	5.21	0.605	.86	−2.67	4.39	0.632
**Living Situation**												
Co-residing^b^	1.55	−4.81	7.91	0.631	0.51	−5.71	6.73	0.871	1.55	−5.62	8.72	0.670
**Interaction: time##co-residency**^**c**^												
Co-residing^b^	6.85	0.39	13.31	**0.038**	6.64	0.36	12.93	**0.038**	6.85	0.16	13.54	**0.045**
PwD age	−0.20	−0.49	0.09	0.167	−0.19	− 0.47	0.08	0.168	−0.20	−0.50	0.10	0.195
CG age	0.15	−0.08	0.38	0.200	0.16	−0.06	0.38	0.155	0.15	−0.09	0.39	0.225
**PwD gender**												
Female^d^	−2.64	−6.69	1.40	0.199	−2.03	− 5.98	1.93	0.314	−2.64	−6.55	1.26	0.184
**Cg gender**												
Female^d^	−1.01	−4.83	2.82	0.605	−1.52	−5.26	2.22	0.422	−1.00	−4.81	2.80	0.603
**CG Working**												
No^d^	− 2.52	−7.65	2.60	0.333	−1.24	− 6.29	3.80	0.628	−2.52	− 6.99	1.93	0.265
**Health care services**												
Home nursing service	−0.18	− 0.35	0.01	0.052	−0.14	− 0.31	0.03	0.115	−0.17	−0.29	−0.06	**0.003**
**Clinical variables**												
MMSE^f^	−0.31	− 0.78	0.17	0.203	−0.38	− 0.85	0.08	0.109	−0.30	−0.79	0.17	0.205
PSMS^g^	0.79	0.09	1.50	**0.026**	0.98	0.29	1.66	**0.006**	0.77	0.15	1.44	**0.016**
IADL^h^	0.15	− 0.37	.67	0.571	−0.02	− 0.53	0.49	0.944	0.15	− 0.36	0.66	0.561
**Municipality**^**i**^												
Bergen	−2.83	−6.93	1.26	0.174	−1.90	−5.93	2.11	0.352	−2.83	− 7.46	1.79	0.229
Baerum	−8.05	− 20.18	4.08	0.192	−6.42	−18.27	5.43	0.286	−8.05	−15.29	− 0.81	**0.029**
Additional CG^j^					−7.45	−11.91	− 2.99	**0.001**		–		
AIC		1489.93				1480.43				1480.433		
Prob > F		< 0.001				< 0.001				< 0.001		
R-squared		0.293				0.333				0.293		

*CG* caregiver, *PwD* Person with dementia, *CI* Confidence Interval, applying Ordinary Least Square (OLS) analysis. Model (1) includes the main effect of the lockdown on the informal resource use in days on personal hygiene (ADL) in the last month. Model (2) an extended model, by including multiple caregivers. Model (3) robustness test for core coefficients. *p < .05; **p < .01

^a^National lockdown in Norway came into force 12th of March 2020, reference: pre-lockdown period

^b^Reference: visiting caregiver

^c^Interaction term as “visiting##pre-pandemic” and “co-resident##pandemic”

^d^Reference: male

^e^Reference: caregivers who were working when lockdown came into force

^f^MMSE – Mini-Mental Status Examination, at the trial inclusion (range 0–30)

^g^PSMS - Physical Self-Maintenance Scale (range 6–30)

^h^Instrumental Activities of Daily Living Scale (range 8–31)

^i^Reference: Kristiansand municipality

^j^Reference: no additional caregivers

In addition to the main estimations in Table 5, we ran OLS regression by transforming our dependent variable from levels to differences. Multivariate regression analysis presented in table A1 suggests that lower IADL function (β = − 2.51, CI, − 4.32 - -0.69, *P* = 0.007) and higher cognitive function (β = − 0.64, CI, − 1.26 – 0.02, *P* = 0.044) were associated with formal care reduction during the lockdown. Thus, as the main estimations and the additional OLS regressions showed, demographic variables of both PwD and caregivers are not significant when determining formal and informal care after lockdown implementations. In contrast, we see that co-residency, as well as clinical variables such as MMSE are crucial factors in determining access to care during lockdown.

## Discussion

The aim of this prospective was investigate the immediate consequences of the COVID-19 lockdown on resource utilization among home-dwelling PwD in Norway. As expected, the ratio between formal and informal caregiving changed significantly during the restrictions. Regardless of their living situation, almost 70% of relatives of a PwD reported an increase in their care responsibilities. The results also confirm that informal care provision is dissimilar across various groups of caregivers and co-residing relatives provided more care than visiting caregivers, especially in personal hygiene tasks. However, formal homecare services did not identify and prioritize PwD who were living alone. These individuals received both less formal and informal care compared to those who lived in a family setting. The lack of prioritization may widen the gap between those who have more care support and those who have less. This observation can be thought of as example of “the Matthew effect”, which refers to the idea that both advantages and disadvantages tend to accumulate, which in this case results in continuously widening differences in living situations. These findings are of key importance for stakeholders and policymakers so that they can better plan support for home-dwelling PwD in the upcoming post-pandemic period and in future crises. Not only does society benefit from better planning, but because dementia can strike anyone, we as individuals might also benefit from well-planned, dementia-friendly independent and safe living at home when we get older.

Co-residing caregivers (mostly spouses) are particularly affected and prone to care overburden by the pandemic lockdown and the sudden disruption in formal care. In our study, 22% of PwD or their caregivers self-cancelled municipal care services. Reasons for this reaction may be found in a study by West et al. [31], which qualitatively explored effects of the COVID-19 pandemic on PwD (*N* = 15) and their family caregivers in the black, Asian and minority ethnic (BAME) communities in the UK. The study identified eight themes (fear and anxiety, food and eating, isolation and identity, and community and social relationships) that were most pertinent to their experiences of community dementia care and COVID-19’s impact on their daily lives. A study from Israel by Werner et al. [32] demonstrates that co-residing with PwD, feelings of burden and low income level were the factors associated with caregivers’ forgone care from general practitioners and medical specialists. Fear and anxiety (in the form of a desire to avoid infection) may have contributed to the increase in informal care provided by co-residing caregivers and the reduction in formal care delivered by homecare services in Norway. This is supported by Giebel et al., showing that the task of risk management decision making for paid home care during the pandemic are challenging [33].

Systematic review by Rosenwohl-Mack et al., including studies conducted pre-pandemic from five continents, demonstrated mixed findings of the magnitude of home-based care use among PwD living alone. They suggest that varying availability of services, policies, and state budgets might be the reason [7]. Consistent with findings from another Norwegian study by Moholt et al., co-habiting PwD receive less formal care suggesting that spouses perform tasks that otherwise would have been performed by health care services [34]. We propose that due to lockdown, the substitution of formal care by informal caregivers is greater than it was before. However, PwD who were living alone during COVID-19 seem to be in more vulnerable situations. Our data suggest that during the lockdown formal care was reduced to an even larger extent in this group, but for these people, the formal care was not replaced by informal care. The lack of guidelines about what should be done in this situation has a critical significance for all stakeholders and may increase the prevalence of adverse events (e.g., potential inappropriate medication, falls, loneliness, depression, and acceleration of dementia) [35].

A recent national report evaluating the first weeks of the COVID-19 lockdown described the critical situation in Norwegian hospitals and nursing homes and the lack of medical personnel, routines in medication distribution, and safety equipment such as face-masks and disinfectants [36]. The situation for home-dwelling older adults with chronic complex conditions (including dementia and their caregivers) were not mentioned in this report despite the fact that of the 70,000 PwD living at home, probably 30,000 of them are alone.

A few studies have explored how the pandemic has affected caregivers to PwD and how the service utilization has degenerated when lockdown started. An Argentinian study [37] demonstrated that anxiety, depression, and insomnia were more prevalent among people with mild dementia than those with severe dementia. Further, pandemic restrictions increased caregiver distress independently of the dementia stage. In severe cases of dementia, formal care disruption was especially worrying [38]. A recent review by Dawson et al. [39] shows differences between countries in how formal care was tailored during the COVID-19 pandemic. Contrary to our results, authors demonstrate that in some countries (e.g., Germany, China and Australia) the service delivery was modified, and (contrary to our results) there was an *increase* in home-based or remote support care in several countries, to compensate for closed daycare centers [39]. An Austrian study demonstrated that closing daycare shifted care resources from formal to informal caregivers [40].

Our study demonstrates the need for a more comprehensive plan of care continuity, better monitoring of care determinants, and enhanced support of informal caregivers. High informal care is associated with caregiver demographics such as higher age and female gender [25, 41], being employed [17], living together with PwD [41], and PwD clinical patient factors such as severity of dementia [42], increased prevalence of BPSD [41, 43, 44], and decrease in physical activities [17, 45].

As the burden of dementia rises worldwide, the need for care will continue to rise, putting enormous additional pressures on primary healthcare systems and family caregivers [46]. Currently in Norway, every 8th inhabitant works in the healthcare sector; by 2050, it is estimated that one in every three will work in this sector. If the global numbers of healthcare workers do not increase, elder care will suffer from a nursing shortage.

### Strengths and limitations

A strength of the PAN.DEM study is the prospective design and the use of the validated RUD instrument, which allows us to contrast our results with comparable data from other populations with dementia. Recall bias and data inaccuracy have been minimized by collecting data from the same caregivers in short periods.

There are some limitations to consider. The sample size is small, and it is hard to demonstrate causal relationships; however, the sample is well-balanced across relationships, gender, and age among PwD and their caregivers. This is the first study to show associations of resource use before and after the COVID-19 pandemic lockdown in any Nordic country. We measured informal care during the lockdown in days but formal care in hours. Still, this limitation did not invalidate the goal of our study, which was to assess whether COVID-19 restrictions affected co-residing and visiting caregivers differently.

## Conclusion

During the COVID-19 lockdown, the intensity of informal care increased significantly among co-residing caregivers. However, restrictions in informal care imposed by the lockdown were less compensated among those living alone with PwD who received fewer hours of formal care and fewer visits from their relatives. This study aims to encourage stakeholders for better identification and prioritization of these individuals in the forthcoming post-pandemic period and in any future epidemic crisis.

## Data Availability

The dataset used in this study is available upon request from the corresponding author or primary investigator.

## Appendix

**Table 6:** Association between socio-demographic and clinical determinants and change in formal and informal care, N = 105

Covariates variables	Total formal care time^a,b^				Home nursing service^b^			
	Coef.	95% CI lower	95% CI upper	*P*	Coef.	95% CI lower	95% CI upper	*P*
Co-residency	−7.42	−26.47	11.66	0.441	5.50	−1.40	12.40	0.116
PwD age	−0.24	−1.28	0.79	0.640	0.12	−0.25	0.49	0.519
CG age	0.59	−0.24	1.43	0.163	0.03	−0.27	0.33	0.849
PwD gender, female	−2.65	−17.24	11.94	0.719	−1.67	−6.91	3.59	0.530
CG gender, female	−3.30	−17.42	10.80	0.642	−1.44	−6.47	3.59	0.571
CG not working	3.70	−14.53	21.94	0.687	−1.38	−7.94	5.18	0.676
MMSE	−0.74	−2.45	0.98	0.396	−0.64	−1.26	− 0.02	**0.044**
IADL	−2.51	−4.32	−0.69	**0.007**	−0.72	−1.62	0.17	0.112
PSMS	0.22	−2.26	2.71	0.858	−0.29	−0.95	0.36	0.375
Prob > F	0.031				0.042			
R-squared	0.190				0.177			
n	94				96			

*N* = total sample, *n* = number of patients, *CG* caregiver, *PwD* Person with dementia, *CI* Confidence Interval

^a^Home nursing, home help, daycare center

^b^Hours in a month

^c^MMSE – Mini-Mental Status Examination, at the trial inclusion (range 0–30)

^d^PSMS - Physical Self-Maintenance Scale (range 6–30)

^e^Instrumental Activities of Daily Living Scale (range 8–31)

## References

[1] Husebø BS, Berge LI (2020). Intensive medicine and nursing home care in times of SARS CoV-2. A Norwegian perspective. Am J Geriatr Psychiatry.

[2] Organisation for European Economic Co-operation (2020). Beyond Containment: Health systems responses to COVID-19 in the OECD.

[3] Pashakhanlou AH. Sweden's coronavirus strategy: The Public Health Agency and the sites of controversy. World Medical & Health Policy. 2021. 10.1002/wmh3.449. 10.1002/wmh3.449 PMC8242624 34226854

[4] Wong SYS, Zhang D, Sit RWS, Yip BHK, RY-n C, CKM W (2020). Impact of COVID-19 on loneliness, mental health, and health service utilisation: a prospective cohort study of older adults with multimorbidity in primary care. Br J Gen Pract.

[5] Askim J, Bergström T. Between lockdown and calm down. Comparing the COVID-19 responses of Norway and Sweden. Local Gov Stud. 2021:1–21. 10.1080/03003930.2021.1964477.

[6] Michalowsky B, Hoffmann W, Bohlken J, Kostev K (2020). Effect of the COVID-19 lockdown on disease recognition and utilisation of healthcare services in the older population in Germany: a cross-sectional study. Age Ageing.

[7] Rosenwohl-Mack A, Dubbin L, Chodos A, Dulaney S, Fang M-L, Merrilees J (2021). Use of Services by People Living Alone With Cognitive Impairment: A Systematic Review. Innov Aging.

[8] Topriceanu C-C, Wong A, Moon JC, Hughes AD, Bann D, Chaturvedi N, Patalay P, Conti G, Captur G (2021). Evaluating access to health and care services during lockdown by the COVID-19 survey in five UK national longitudinal studies. BMJ Open.

[9] Gjøra L, Heine Strand B, Bergh S, Borza T, Brækhus A, Engedal K (2021). Current and Future Prevalence Estimates of Mild Cognitive Impairment, Dementia, and Its Subtypes in a Population-Based Sample of People 70 Years and Older in Norway: The HUNT Study. J Alzheimers Dis.

[10] Ringard Å, Sagan A, Sperre Saunes I, Lindahl AK, World Health Organization (2013). Norway: health system review. Health Syst Transit.

[11] Vossius C, Selbæk G, Ydstebø A, Benth J, Godager G, Lurås H (2015). Ressursbruk Og Sykdomsforløp Ved Demens (REDIC) [resource use and disease course in dementia (REDIC)].

[12] Boutoleau-Bretonnière C, Pouclet-Courtemanche H, Gillet A, Bernard A, Laure Deruet A, Gouraud I, et al. The effects of confinement on neuropsychiatric symptoms in Alzheimer's disease during the COVID-19 crisis. J Alzheimers Dis. 2020;(Preprint):1–7. 10.3233/JAD-200604. 10.3233/JAD-200604 PMC9988367 32568211

[13] Cagnin A, Di Lorenzo R, Marra C, Bonanni L, Cupidi C, Laganà V (2020). Behavioral and psychological effects of coronavirus disease-19 quarantine in patients with dementia. Front Psychiatry.

[14] Giebel C, Cannon J, Hanna K, Butchard S, Eley R, Gaughan A, Komuravelli A, Shenton J, Callaghan S, Tetlow H, Limbert S, Whittington R, Rogers C, Rajagopal M, Ward K, Shaw L, Corcoran R, Bennett K, Gabbay M (2021). Impact of COVID-19 related social support service closures on people with dementia and unpaid carers: a qualitative study. Aging Ment Health.

[15] Mazzi MC, Iavarone A, Musella C, De Luca M, de Vita D, Branciforte S (2020). Time of isolation, education and gender influence the psychological outcome during COVID-19 lockdown in caregivers of patients with dementia. Eur Geriatr Med.

[16] Giebel C, Lord K, Cooper C, Shenton J, Cannon J, Pulford D, Shaw L, Gaughan A, Tetlow H, Butchard S, Limbert S, Callaghan S, Whittington R, Rogers C, Komuravelli A, Rajagopal M, Eley R, Watkins C, Downs M, Reilly S, Ward K, Corcoran R, Bennett K, Gabbay M (2021). A UK survey of COVID-19 related social support closures and their effects on older people, people with dementia, and carers. Int J Geriatr Psychiatry.

[17] Michalowsky B, Thyrian JR, Eichler T, Hertel J, Wucherer D, Flessa S, Hoffmann W (2016). Economic analysis of formal care, informal care, and productivity losses in primary care patients who screened positive for dementia in Germany. J Alzheimers Dis.

[18] Bakker C, de Vugt ME, van Vliet D, Verhey FR, Pijnenburg YA, Vernooij-Dassen MJ (2013). The use of formal and informal care in early onset dementia: results from the NeedYD study. Am J Geriatr Psychiatry.

[19] Gedde MH, Husebo BS, Erdal A, Puaschitz NG, Vislapuu M, Angeles RC, Berge LI (2021). Access to and interest in assistive technology for home-dwelling people with dementia during the COVID-19 pandemic (PAN.DEM). Int Rev Psychiatry.

[20] Husebo BS, Allore H, Achterberg W, Angeles RC, Ballard C, Bruvik FK, Fæø SE, Gedde MH, Hillestad E, Jacobsen FF, Kirkevold Ø, Kjerstad E, Kjome RLS, Mannseth J, Naik M, Nouchi R, Puaschitz N, Samdal R, Tranvåg O, Tzoulis C, Vahia IV, Vislapuu M, Berge LI (2020). LIVE@ home. Path-innovating the clinical pathway for home-dwelling people with dementia and their caregivers: study protocol for a mixed-method, stepped-wedge, randomized controlled trial. Trials.

[21] Mitchell AJ (2017). The Mini-mental state examination (MMSE): update on its diagnostic accuracy and clinical utility for cognitive disorders.

[22] Reisberg B (1988). Functional assessment staging (FAST). Psychopharmacol Bull.

[23] Wimo A, Gustavsson A, Jonsson L, Winblad B, Hsu MA, Gannon B (2013). Application of resource utilization in dementia (RUD) instrument in a global setting. Alzheimers Dement.

[24] Wimo A, Jonsson L, Zbrozek A (2010). The resource utilization in dementia (RUD) instrument is valid for assessing informal care time in community-living patients with dementia. J Nutr Health Aging.

[25] Wimo A, Gauthier S, Prince M. Global estimates of informal care. In: World Alzheimer report. London: Alzheimer's disease international (ADI) and Karolinska Institute; 2018.

[26] Arevalo-Rodriguez I, Smailagic N, Roqué i Figuls M, Ciapponi A, Sanchez-Perez E, Giannakou A, et al. Mini-mental state examination (MMSE) for the detection of Alzheimer's disease and other dementias in people with mild cognitive impairment (MCI). Cochrane Database Syst Rev. 2015;3. 10.1002/14651858.CD010783.pub2. 10.1002/14651858.CD010783.pub2 PMC6464748 25740785

[27] Lawton MP, Brody EM (1969). Assessment of older people: self-maintaining and instrumental activities of daily living. Gerontologist.

[28] Brody E, Lawton M (1988). Physical self-maintenance scale (PSMS).

[29] Schneider LS, Olin JT, Doody RS, Clark CM, Morris JC, Reisberg B (1997). Validity and reliability of the Alzheimer's disease cooperative study-clinical global impression of change (ADCS-CGIC).

[30] Razali NM, Wah YB (2011). Power comparisons of shapiro-wilk, kolmogorov-smirnov, lilliefors and Anderson-darling tests. J Stats Modeling Analytic.

[31] West E, Nair P, Barrado-Martin Y, Walters KR, Kupeli N, Sampson EL, Davies N (2021). Exploration of the impact of the COVID-19 pandemic on people with dementia and carers from black and minority ethnic groups. BMJ Open.

[32] Werner P, Tur-Sinai A, AboJabel H (2021). Examining dementia family Caregivers' forgone Care for General Practitioners and Medical Specialists during a COVID-19 lockdown. Int J Environ Res Public Health.

[33] Giebel C, Hanna K, Cannon J, Eley R, Tetlow H, Gaughan A, Komuravelli A, Shenton J, Rogers C, Butchard S, Callaghan S, Limbert S, Rajagopal M, Ward K, Shaw L, Whittington R, Hughes M, Gabbay M (2020). Decision-making for receiving paid home care for dementia in the time of COVID-19: a qualitative study. BMC Geriatr.

[34] Moholt J-M, Friborg O, Blix BH, NJD H (2018). Factors affecting the use of home-based services and out-of-home respite care services: A survey of family caregivers for older persons with dementia in Northern Norway. Dementia (London).

[35] Livingston G, Sommerlad A, Orgeta V, Costafreda SG, Huntley J, Ames D, Ballard C, Banerjee S, Burns A, Cohen-Mansfield J, Cooper C, Fox N, Gitlin LN, Howard R, Kales HC, Larson EB, Ritchie K, Rockwood K, Sampson EL, Samus Q, Schneider LS, Selbæk G, Teri L, Mukadam N (2017). Dementia prevention, intervention, and care. Lancet.

[36] Comission C (2021). Koronakommisjonens rapport (NOU).

[37] Cohen G, Russo MJ, Campos JA, Allegri RF (2020). COVID-19 epidemic in Argentina: worsening of behavioral symptoms in elderly subjects with dementia living in the community. Front Psychiatry.

[38] Cohen G, Russo MJ, Campos JA, Allegri RF (2020). Living with dementia: increased level of caregiver stress in times of COVID-19. Int Psychogeriatr.

[39] Dawson WD, Ashcroft EC, Lorenz-Dant K, Comas-Herrera A (2020). Mitigating the impact of the COVID-19 outbreak: a review of international measures to support community-based care.

[40] Rodrigues R, Simmons C, Schmidt AE, Steiber N. Care in times of COVID-19: the impact of the pandemic on informal caregiving in Austria. Eur J Ageing. 2021:1–11. 10.31235/osf.io/bj3fk. 10.1007/s10433-021-00611-z PMC7952831 33727905

[41] Farré M, Haro JM, Kostov B, Alvira C, Risco E, Miguel S, Cabrera E, Zabalegui A (2016). Direct and indirect costs and resource use in dementia care: a cross-sectional study in patients living at home. Int J Nurs Stud.

[42] Wimo A, Reed CC, Dodel R, Belger M, Jones RW, Happich M, Argimon JM, Bruno G, Novick D, Vellas B, Haro JM (2013). The GERAS study: a prospective observational study of costs and resource use in community dwellers with Alzheimer's disease in three European countries - study design and baseline findings. J Alzheimers Dis.

[43] Costa N, Wübker A, De Mauléon A, Zwakhalen SM, Challis D, Leino-Kilpi H (2018). Costs of care of agitation associated with dementia in 8 European countries: results from the RightTimePlaceCare study. J Am Med Direct Assoc.

[44] Teipel SJ, Thyrian JR, Hertel J, Eichler T, Wucherer D, Michalowsky B, Kilimann I, Hoffmann W (2015). Neuropsychiatric symptoms in people screened positive for dementia in primary care. Int Psychogeriatr.

[45] Gustavsson A, Jonsson L, Rapp T, Reynish E, Ousset P, Andrieu S (2010). Differences in resource use and costs of dementia care between European countries: baseline data from the ICTUS study. J Nutr Health Aging.

[46] Nichols E, Szoeke CE, Vollset SE, Abbasi N, Abd-Allah F, Abdela J (2019). Global, regional, and national burden of Alzheimer's disease and other dementias, 1990-2016: a systematic analysis for the global burden of disease study 2016. Lancet Neurol.

[47] Wolford B (2020). Data Protection Impact Assessment (DPIA). How to conduct a Data Protection Impact Assessment.

